# Identification of a Novel Wnt Antagonist Based Therapeutic and Diagnostic Target for Alzheimer’s Disease Using a Stem Cell-Derived Model

**DOI:** 10.3390/bioengineering10020192

**Published:** 2023-02-02

**Authors:** Manasi Patil, Naisarg Gamit, Arun Dharmarajan, Gautam Sethi, Sudha Warrier

**Affiliations:** 1 Division of Cancer Stem Cells and Cardiovascular Regeneration, Manipal Institute of Regenerative Medicine, Manipal Academy of Higher Education (MAHE), Bangalore 560 065, India; 2 Department of Biomedical Sciences, Faculty of Biomedical Sciences and Technology, Sri Ramachandra Institute of Higher Education and Research, Chennai 600 116, India; 3 School of Human Sciences, Faculty of Life and Physical Sciences, The University of Western Australia, Perth, WA 6009, Australia; 4 Curtin Medical School, Curtin University, Perth, WA 6102, Australia; 5 Department of Pharmacology, Yong Loo Lin School of Medicine, National University of Singapore, Singapore 117 600, Singapore; 6 Cuor Stem Cellutions Pvt Ltd., Manipal Institute of Regenerative Medicine, Manipal Academy of Higher Education (MAHE), Bangalore 560 065, India

**Keywords:** Alzheimer’s disease, amyloid beta, Wnt pathway, Wnt antagonism, stem cell model, neuroprotection

## Abstract

Currently, all the existing treatments for Alzheimer’s disease (AD) fail to stall progression due to longer duration of time between onset of the symptoms and diagnosis of the disease, raising the necessity of effective diagnostics and novel treatment. Specific molecular regulation of the onset and progression of disease is not yet elucidated. This warranted investigation of the role of Wnt signaling regulators which are thought to be involved in neurogenesis. The AD model was established using amyloid beta (Aβ) in human mesenchymal stem cells derived from amniotic membranes which were differentiated into neuronal cell types. In vivo studies were carried out with Aβ or a Wnt antagonist, AD201, belonging to the sFRP family. We further created an AD201-knockdown in vitro model to determine the role of Wnt antagonism. BACE1 upregulation, ChAT and α7nAChR downregulation with synapse and functionality loss with increases in ROS confirmed the neurodegeneration. Reduced β-catenin and increased AD201 expression indicated Wnt/canonical pathway inhibition. Similar results were exhibited in the in vivo study along with AD-associated behavioural and molecular changes. AD201-knockdown rescued neurons from Aβ-induced toxicity. We demonstrated for the first time a role of AD201 in Alzheimer’s disease manifestation, which indicates a promising disease target and biomarker.

## 1. Introduction

According to the World Health Organization (WHO) in 2019, nearly 50 million people have dementia, and Alzheimer’s disease (AD) contributes to 60–70% of total dementia cases. AD is characterized by neuronal loss, appearance of amyloid plaques and neurofibrillary tangles followed by progressive cognitive decline [1]. A characteristic feature of AD is the accumulation of amyloid-beta (Aβ) protein caused by proteolysis of amyloid precursor protein (APP) [2], which in a normal brain is cleared by neprilysin and ApoE [3]. However, in the AD brain, abnormal rise in Aβ accumulation results in an imbalance between its accumulation and clearance and plaques are formed. Mutations in genes such as APP, Tau and presenilin-1 causing higher Aβ production and failure in its clearance are observed in familial AD and sporadic AD, respectively. 

Alzheimer’s disease has caused a substantial burden on medical treatment as the current treatments only improve the symptoms without altering the disease progression. As AD is a complex multifactorial disease, it is difficult to develop a novel strategy to alleviate the disease without appropriate knowledge of disease progression and interplay of multiple signaling pathways involved in it. Several groups have reported involvement of Wnt signaling in AD pathogenesis. However, the exact role of the Wnt signaling pathway in AD still remains unknown. Previous studies have demonstrated that overexpression of β-catenin and inhibition of GSK3-β, decreased the Aβ levels and activity of β-APP cleaving enzyme (BACE1) [4], whereas overexpression of Wnt antagonist Dkk1 caused Aβ induced neurotoxicity [5,6].

The available in vitro AD models use transgenic models, cancer cell lines such as SH-SY5Y, immortalized cell lines and primary neuron cultures [7,8,9]. In earlier studies, mutations in APP, Tau and presenilin genes were introduced in rodent models. Additionally, invertebrate models from drosophila and *C. elegans* were also used [10]. However, failure in recapitulation of the pathology limits the use of both the models. For example, the mouse model of AD does not closely mimic the disease since the rodent amyloid-β peptide differs from those in humans and therefore do not aggregate. Additionally, in cell line-based models, axons and synaptic loss is not observed as they lack neuronal morphology and neuron-specific markers. Moreover, live and functional adult neurons are not readily available. Therefore, in vitro and in vivo AD models that mimic the progressive neurodegeneration and loss of cognition with amyloid plaques and neurofibrillary tangles are challenging.

Several attempts have been made to model AD from stem cell-derived neurons to mimic the disease in terms of fibrillary deposits, loss of neurons and cellular interactions. Stem cell-derived models of Alzheimer’s disease include use of human induced pluripotent stem cells (hiPSCs) and human embryonic stem cells (hESCs) [7,11], which involve extensive genetic manipulations and risk of damaging the pre-implantation embryo. In our study, we used amniotic membrane-mesenchymal stem cells (AM-MSC) obtained from perinatal discard to develop a model mimicking the disease and further investigate the Wnt signaling pathway modulation in AD. The advantage of using AM-MSC lies in the fact that these are adult stem cells, but mirror more of an embryonic multi-potency [12]. We differentiated AM-MSC into neurons and induced AD-like pathology with aggregated Aβ protein. In this model, it was observed that the cells underwent neurodegeneration and also exhibited increased expression of BACE1 and reduced levels of acetylcholine. Further, the changes in Wnt signaling after induction of AD-like pathology in both in vitro and in vivo models along with neuroprotection provided by commercially available drugs were studied.

## 2. Results

### 2.1. Isolation, Culture and Characterization of AM-MSC

**Figure 1:**
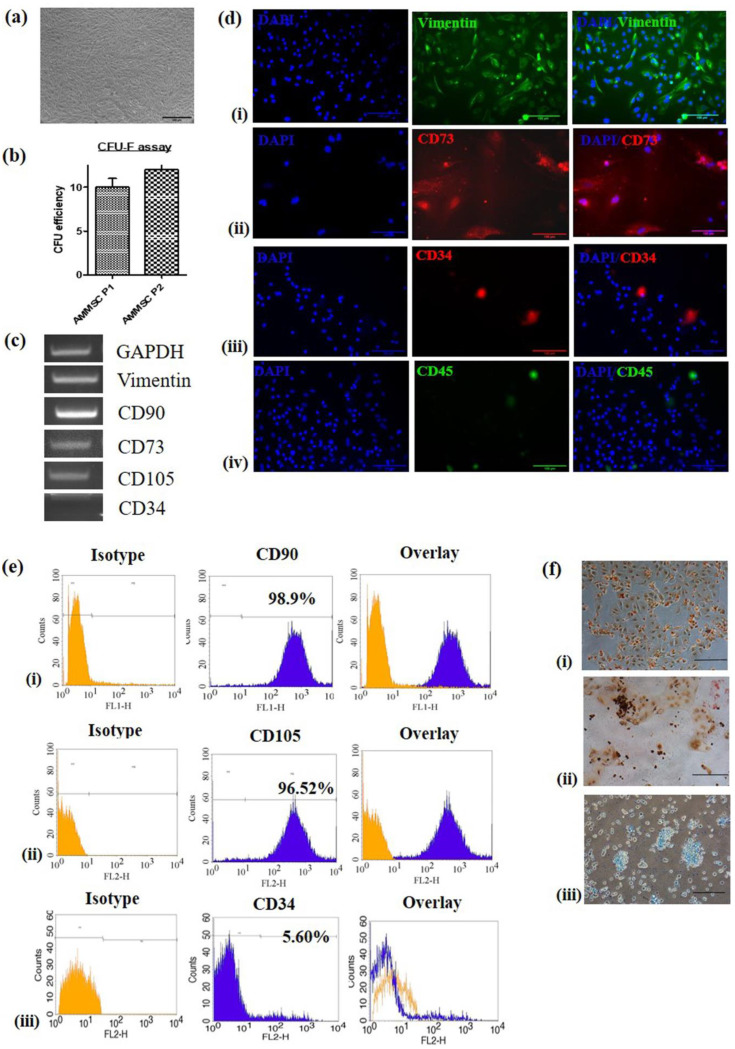
Isolation of amniotic-membrane mesenchymal stem cells (AM-MSC) and its characterization (**a**) Photomicrographs of AM-MSC at passage 2 (4× magnification, scale bar = 200 µm). (**b**) Colony formation ability of AM-MSC at passage 1 and 2. (**c**) Analysis of MSC markers vimentin, CD90, CD73 and CD105 and negative marker CD34 by RT-PCR. (**d**) Immunofluorescence staining revealing the presence of cytoskeletal marker (**i**) vimentin and specific surface markers (**ii**) CD73, (**iii**) CD34 and (**iv**) CD45 (20× magnification, scale bar = 100 µm). (**e**) Analysis of MSC surface markers by flow cytometry showing (**i**) 98.9% cells positive for CD90, (**ii**) 96.52% cells positive for CD 105, (**iii**) 94.28% cells negative for CD 34. (**f**) Photomicrographs showing trilineage differentiation in AM-MSC, (**i**) adipocytes stained with oil red ‘O’ showing oil droplets stained red, (**ii**) 2% Alizarin red staining performed on osteocytes showing the calcium deposits-stained reddish brown, (**iii**) Alcan blue staining performed on chondrocytes showing polysaccharides stained blue (20× magnification, scale bar = 100 µm).

AM-MSC adhered to the culture dish, displayed polygonal morphology and upon further culturing, showed typical spindle shaped morphology (Figure 1a). MSC at passage 2 had greater CFU-F efficiency than that at passage1 (Figure 1b). RT-PCR showed AM-MSC were positive for MSC markers, vimentin, CD90, CD73 and CD105, but were negative for CD34 (Figure 1c, Supplementary Data S1-a). Immunofluorescence showed cells expressing vimentin and CD73 but lacking expression of CD34 and CD45 (Figure 1d). In addition, flow cytometry showed AM-MSC positive for CD90 (98.9%), CD105 (96.5%) and negative for CD34 (Figure 1e(i–iii)). All the results showed that AM-MSC cultures fulfilled ISCT criteria. 

### 2.2. Trilineage Differentiation

As per the ISCT criteria defining MSC, these cells must differentiate to adipocytes, osteoblasts and chondroblasts in vitro. Multi-potential capacity of AM-MSC was established by inducing the cells to adipogenic, osteogenic and chondrogenic differentiation. AM-MSC showed accumulation of oil droplets following adipogenic induction (Figure 1f(i)). Osteogenic differentiation was confirmed by the presence of brown coloured calcium deposits with Alizarin red (Figure 1f(ii)). Alcan Blue, which stains glycosaminoglycans, confirmed the chondrogenic lineage differentiation (Figure 1f(iii)). 

### 2.3. Differentiation of AM-MSCs into Neuronal Cells

**Figure 2:**
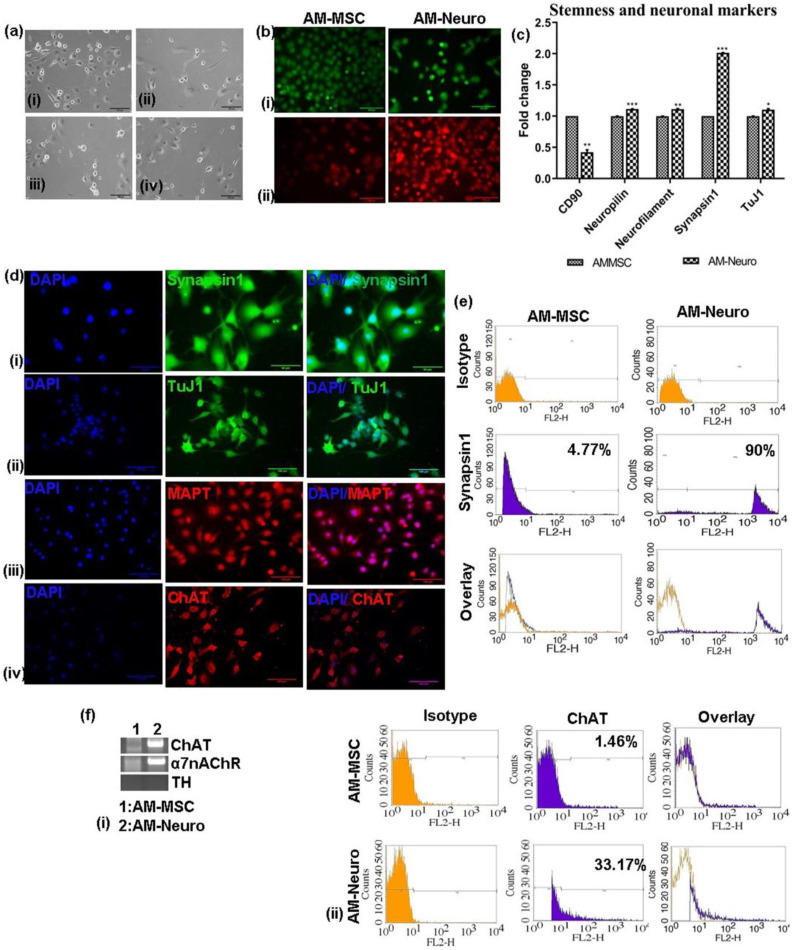
Differentiation of AM-MSC into neural cell types and its characterization. (**a**) Photomicrographs of neural cell types derived from AM-MSC upon culturing in induction medium, (**i**) neurons, (**ii**) microglia, (**iii**) oligodendrocyte, (**iv**) astrocytes (20× magnification, scale bar = 100 µm). (**b**) Microscopic images of cells stained with (**i**) Calcein-AM staining the intracellular esterases, (**ii**) TMRE staining the active mitochondria (20× magnification, scale bar = 100 µm). (**c**) qRT-PCR analysis showing downregulation of MSC marker-CD90 and upregulation of neural markers: neuropilin, neurofilament, synapsin1 and tuj1 in the differentiated cells. Data are represented as mean with SEM (*n* = 3), * *p* < 0.05, ** *p* < 0.01 *** *p* < 0.001. (**d**) Immunofluorescence analysis performed on AM-Neuro cells for neural markers (**i**) synapsin1, (**ii**) tuj1, (**iii**) MAPT and (**iv**) ChAT (20× magnification, scale bar = 100 µm). (**e**) Flow cytometry analysis of the AM-MSC and AM-Neuro cells showing 90% of cells positive for synapsin1 (AM-Neuro). (**f**) (**i**) Gene expression analysis by RT-PCR for cholinergic marker ChAT, a7nAChR and dopaminergic marker, TH (**ii**) Flow cytometry analysis showing 33.17% of AM-Neuro cells positive for ChAT.

We analyzed morphological changes in induced AM-MSC 16–20 h post-induction wherein the change in their spindle, flat morphology to neuron-like cells and a lesser number of oligodendrocytes and astrocytes was observed (Figure 2a(i–iv)). Cell viability of AM-Neuro cells was not affected upon inducing differentiation (Figure 2b(i)) and also, TMRE staining on AM-Neuro cells showed that they had higher number of active mitochondria compared to AM-MSC (Figure 2b(ii)).

Gene expression analysis of AM-Neuro cells indicated a significant decrease in the expression of MSC marker, CD90, in AM-Neuro cells and increase in expression of mature neuronal markers, such as neuropilin, neurofilament, synapsin1 and tuj1 (Figure 2c). Further, immunofluorescence analysis confirmed the expression of mature neuronal markers such as synapsin1, tuj1 and MAPT (Figure 2d(i–iii)). The presence of synapsin1 positive cells was further confirmed by flow cytometry analysis which revealed that the cells were positive for synapsin1 (90%) (Figure 2e).

To analyze the presence of cholinergic markers, we studied gene expression of differentiated cholinergic neuron marker- ChAT, acetylcholine receptor- α7nAChR and dopaminergic neuron marker- TH. The results showed higher expression of ChAT and α7nAChR in AM-Neuro cells, while showing no expression of TH gene (Figure 2f(i), Supplementary Data S1-b). Expression of ChAT was further confirmed by immunofluorescence (Figure 2d(iv)) and flow cytometry (Figure 2f(ii)). 

### 2.4. AD-like Pathology on Treatment with Aβ_1–42_

**Figure 3:**
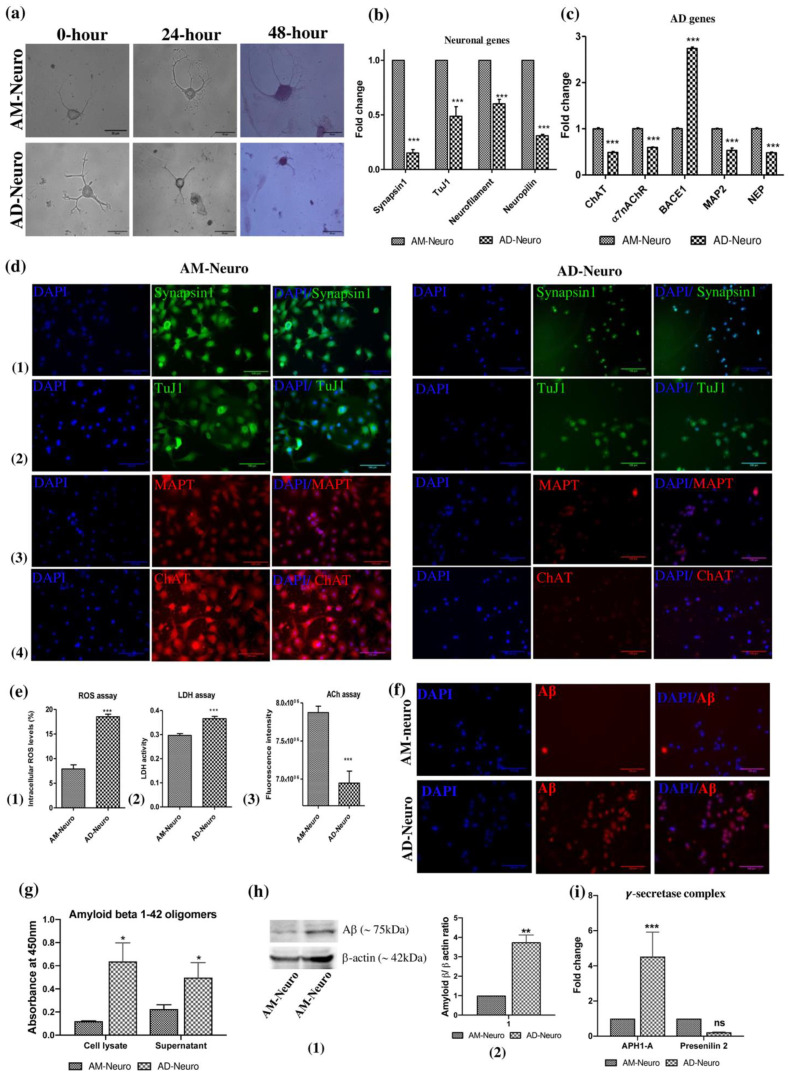
Induction of neurodegeneration was carried out using Aβ_1–42_. (**a**) Photomicrographs of cells undergoing degeneration on treatment with Aβ_1–42_ (AM-Neuro and AD-Neuro cells stained with Giemsa, 40× magnification, scalebar = 50 µm). (**b**) Gene expression by qRT-PCR showing downregulation of neuronal genes synapsin1, tuj1, neurofilament and neuropilin. (**c**) qRT-PCR analysis for ChAT, α7nAChR, BACE1, MAP2 and NEP. Data are represented as mean with SEM (*n* = 3), * *p* < 0.05, ** *p* < 0.01, *** *p* < 0.001. (**d**) Immunofluorescence images of AM-Neuro (left) and AD-Neuro cells (right) showing expression of neuronal markers: (**1**) synapsin1, (**2**) tuj1, (**3**) MAPT and (**4**) ChAT (scale bar = 100 µm). (**e**) Graphs representing increase in (**1**) intracellular ROS, (**2**) LDH activity and (**3**) decrease in ACh levels in terms of fluorescence intensity. Data are represented as mean with SEM (*n* = 3), ** *p* < 0.01, *** *p* < 0.001. (**f**) Fluorescent images showing Aβ accumulation in AD-Neuro cells compared to AM-Neuro cells (scale bar = 100 µm). (**g**) Graph showing Aβ levels (**h**) Western blot and corresponding densitometric analysis for Aβ levels with endogenous control, β-actin (**i**) PCR data showing upregulation of γ-secretase components- APH1-A and Presenilin 2 in AD-Neuro cells.

We first created the AD model by treating AM-Neuro cells with Aβ_1–42_ and the resultant cells were termed as AD-Neuro cells. Firstly, to observe the putative AD-like phenotype, we performed Giemsa staining and the AD-Neuro cells basically showed a loss of neurites (Figure 3a). Neurodegeneration in AD-Neuro cells was studied by gene analysis which showed significant downregulation in the expression of the neuronal markers synapsin1, tuj1, neurofilament and neuropilin (Figure 3b) and a decrease in expression of functional cholinergic markers such as ChAT and α7nAChR (Figure 3c). Confirmation of AD-specific neurodegeneration was seen by significantly elevated BACE1, reflecting the Aβ induced neuronal stress and AD-associated pathology. We also observed a considerable decrease in the expression of microtubule-associated protein type II (MAP2) and neprilysin (NEP), an amyloid-degrading enzyme. The loss of neuronal structure and function was further confirmed by a reduction in synapsin1, tuj1, MAPT and ChAT by immunofluorescence compared to AM-Neuro cells (Figure 3d). 

We performed ROS assay to examine a potential increase in intracellular ROS levels in AD-Neuro cells that could have been released due to Aβ-induced oxidative stress. We observed that there was indeed approximately a two-fold rise in the intracellular ROS levels in AD-Neuro cells (Figure 3e(1)). Similarly, LDH activity was also found to be higher in the AD-neuro cells suggesting impaired metabolism and loss of cell membrane integrity (Figure 3e(2)). Acetylcholine (ACh) acts as neurotransmitter as well as neuromodulator and is involved in arousal, attention, learning and memory. The ACh decrease due to a deficit in the cholinergic pathway has been associated with AD. We found that the ACh levels dropped significantly in AD-Neuro cells (Figure 3e(3)). We confirmed the presence of aggregated Aβ in AD-Neuro cells by immunocytochemical staining, indirect ELISA and Western blot with anti-amyloid β antibody (Figure 3f–h, Supplementary Data S2)). Gene expression analysis showed significant upregulation of γ-secretase components- Anterior pharynx defective-1 (APH1-A) and Presenilin 2 in the Aβ treated cells (Figure 3i).

### 2.5. Wnt Regulators in AD

**Figure 4:**
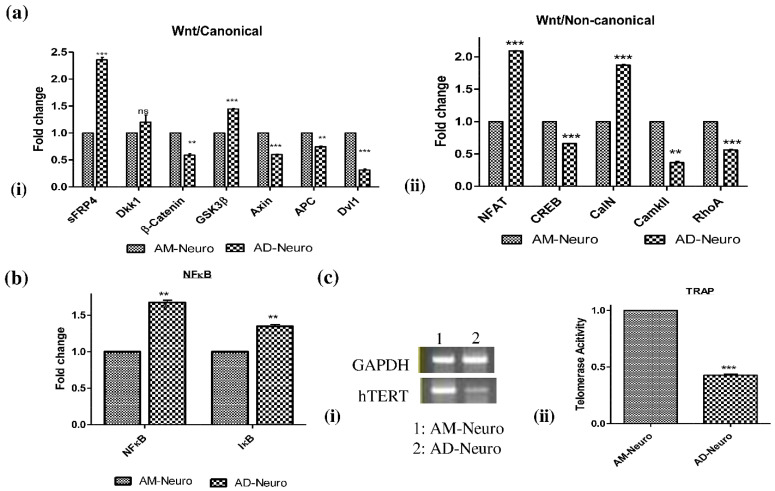
Molecular modulators of Alzheimer’s disease. (**a**) qRT-PCR analysis showing (**i**) downregulation of Wnt/canonical pathway genes- β-catenin, Axin, APC and Dvl1 and increase in expression of GSK3β along with Wnt antagonists, sFRP4 and Dkk1, (**ii**) expression of Wnt/non-canonical pathway genes: NFAT, CREB, CalN, CaMKII and RhoA. (**b**) qRT-PCR data showing increase in NFκB pathway genes, NFκB and IκB in AD-neuro cells. Data are represented as mean with SEM (*n* = 3), ** *p* < 0.01 *** *p* < 0.001. (**c**) (**i**) RT-PCR analysis showing downregulation in hTERT gene in AD-Neuro cells compared to AM-Neuro cells (control), (**ii**) decrease in telomerase activity confirmed by TRAP assay. Data are represented as mean with SEM (*n* = 3), *** *p* < 0.001.

Following confirmation of the loss of neuronal structure, cellular injury and increase in oxidative stress, we further performed gene analysis to understand the status of Wnt signaling in the cells. Wnt signaling is a crucial pathway in nervous system development and is observed to be aberrant in AD. In our AD model, we could observe a clear and significant increase in the gene expression of the inhibitors of Wnt- β catenin pathway such as Glycogen Synthase Kinase 3 (GSK-3β) and Wnt antagonists sFRP4 while the expression of β-catenin was reduced (Figure 4a(i)). However, the increase in expression of another Wnt antagonist, Dkk1, was not significant. Other indicators of the Wnt- β catenin pathway, such as Axin, Adenomatous polyposis coli (APC) and Disheveled (Dvl1) genes were downregulated. In the Wnt-calcium pathway, there was an increase in nuclear factor of activated T cells (NFAT) and calcineurin (CalN) genes whereas CREB and CaMKII expression was reduced in the AD model (Figure 4a(ii)). In the Wnt-PCP signaling axis, Rho-A expression was found to be downregulated (Figure 4a(ii)). Further, we analyzed gene expression of NFκB and IκB to ascertain the Aβ induced activation of inflammatory signaling pathway. Both NFκB and IκB genes were upregulated in the AD model suggesting the activation of NFκB pathway in response to neuronal insult (Figure 4b).

### 2.6. Telomerase Activity

To assess the change in telomeric length associated with neurodegeneration induced by Aβ, we first analyzed the expression of telomerase reverse transcriptase (hTERT) gene. The PCR result revealed that hTERT gene expression was lower in AD-Neuro cells compared to control (AM-Neuro) (Figure 4c(i), Supplementary Data S1-c). We further analyzed the telomerase activity by telomerase repeat amplification protocol (TRAP). We observed that telomerase activity was significantly reduced in the AD-Neuro cells confirming the RT-PCR result (Figure 4c(ii)).

### 2.7. Effect of AD Drugs on the In Vitro AD Model

**Figure 5:**
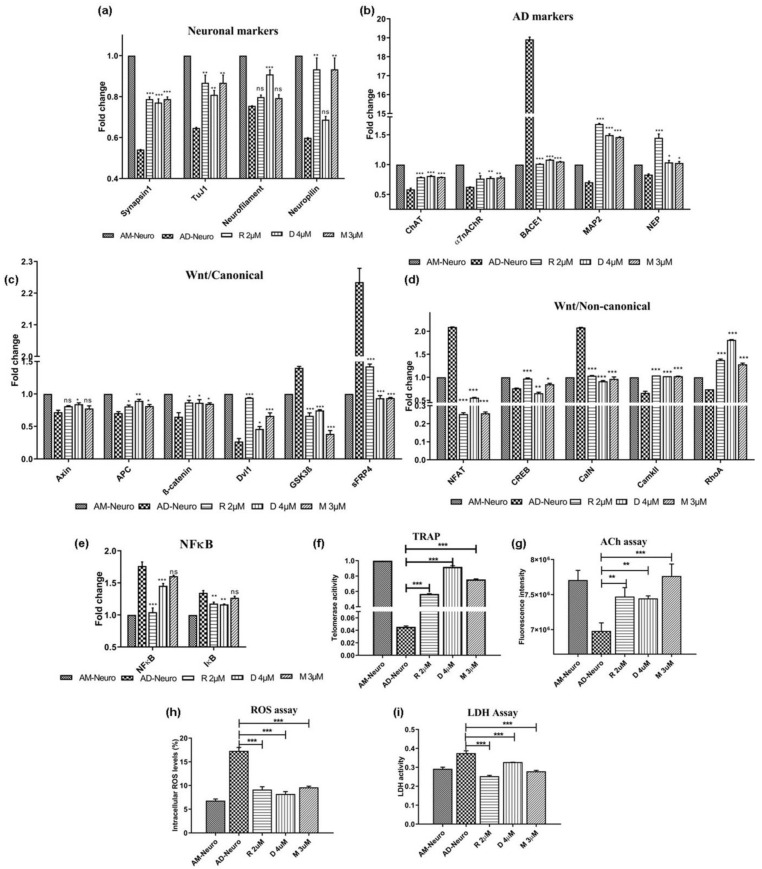
Effect of commercial AD drugs rivastigmine (R), donepezil (D) and memantine (M) on Aβ_1–42_-mediated degeneration in vitro; qRT-PCR analysis of AD-Neuro cells treated with R, D and M for (**a**) neuronal markers: synapsin1, tuj1, neurofilament and neuropilin, (**b**) AD-associated markers: ChAT, α7nAChR, BACE1, MAP2 and NEP, (**c**) Wnt/canonical pathway markers, GSK3β, sFRP4, Axin, APC, β-catenin and Dvl1, (**d**) Wnt/non-canonical pathway markers, NFAT, CREB, CalN, CaMKII and RhoA and (**e**) NFκB and IκB. Data are represented as mean with SEM (*n* = 3), * *p* < 0.05, ** *p* < 0.01 *** *p* < 0.001. (**f**) TRAP assay showing increase in telomerase activity in AD-Neuro cells upon treatment with drugs R, D and M at 2 µM, 4 µM and 3 µM respectively. (**g**) Increase in acetylcholine (ACh) in terms of fluorescence intensity in the cells treated with drugs as compared to AD-Neuro, (**h**) lowering of intracellular ROS production and (**i**) decrease in LDH activity in the drug treated AD-Neuro cells. Data are represented as mean with SEM (*n* = 3), ** *p* < 0.01 *** *p* < 0.001.

Commercially available AD drugs, such as rivastigmine, donepezil and memantine were used in this study to ascertain their effect on the MSC-derived AD model. These drugs were able to successfully rescue the AD-Neuro cells from damage induced by Aβ as observed in the gene expression of synapsin1, tuj1, neurofilament and neuropilin which was more in the drug treated conditions compared to AD-Neuro cells (Figure 5a). In addition, there was also an increase in functional neuronal markers such as ChAT, α7nAChR, MAP2 and NEP genes, whereas BACE1 expression was significantly lowered in the drug-treated cells in comparison to AD-Neuro cells (Figure 5b). Mechanistically, in relation to the Wnt pathway, there was expression of the Wnt- β-catenin pathway as seen by the lowered expression of GSK-3β and sFRP4 and increased Axin, APC, β-catenin and Dvl1 gene expression when compared to AD-Neuro cells (Figure 5c). Further, NFAT and CalN expression were reduced whereas CaMKII and RhoA showed increased levels. These results indicate that the Wnt-calcium pathway was inhibited whereas the Wnt-PCP pathway was activated (Figure 5d). Apart from elevated expression of neuronal markers, we also observed reduction in expression of NFκB and IκB genes in the drug-treated AD-Neuro cells (Figure 5e), suggesting suppression of inflammatory pathway. Telomerase activity was significantly increased in drug-treated cells compared to AD-Neuro cells (Figure 5f). Apart from protecting the neurons from the amyloid induced damage, the drugs significantly raised the acetylcholine (ACh) levels in the cells, indicating maintenance of the functional activity of cholinergic neurons (Figure 5g).

### 2.8. Oxidative Stress and Impaired Metabolism

Earlier, we observed oxidative stress induced by Aβ leading to neurotoxicity (Figure 3e(1)). This effect was lowered upon treatment with rivastigmine, donepezil and memantine (Figure 5h). Intracellular ROS dropped from 17.3% (AD-Neuro) to 9.13%, 8.2% and 9.6% in rivastigmine, donepezil and memantine treated cultures, respectively. Similarly, LDH activity which was found to be very high in Aβ treated cells (0.374), was significantly lowered in all the drug treated cultures (0.253, 0.327 and 0.278 in rivastigmine, donepezil and memantine, respectively) (Figure 5i). 

### 2.9. Aβ_1–42_ and Wnt Antagonist Induced Neurotoxicity in Mice

**Figure 6:**
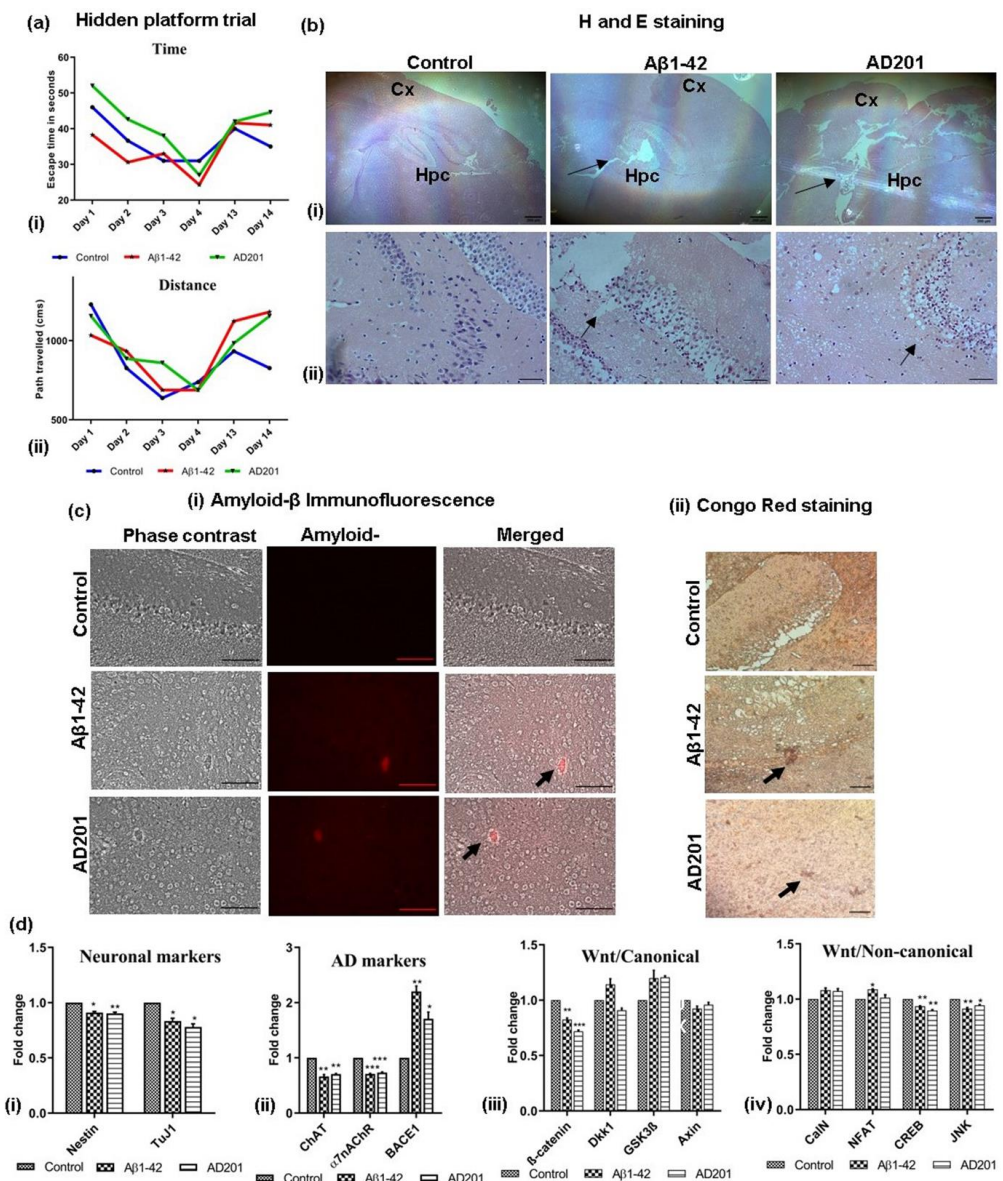
In vivo mouse AD model: comparison of Aβ model with Wnt antagonist, AD201 model. (**a**) Changes in (**i**) escape time and (**ii**) distance traveled by the mice among different groups to reach the hidden platform, (**b**) microscopic images of H and E-stained brain sections showing hippocampal and cortex regions (sham control, Aβ_1–42_ and AD201) at (**i**) 4× (scalebar = 200 µm) and (**ii**) 40× magnification (scalebar = 50 µm); arrows indicating loss of neurons, Hpc-Hippocampus, Cx-Cortex. (**c**) Determination of Aβ_1–42_ deposition in brain sections by (**i**) immunofluorescence and (**ii**) Congo red staining the Aβ_1–42_ deposits (Aβ accumulation indicated by arrow, 20× magnification, scalebar = 100 µm). (**d**) Gene expression analysis by qRT-PCR performed for (**i**) neuronal markers: Nestin, tuj1, (**ii**) AD-associated markers: ChAT, α7nAChR and BACE1, (**iii**) Wnt/canonical pathway genes-β-catenin, Dkk1, GSK3β and Axin; (**iv**) Wnt/non-canonical pathway genes-CalN, NFAT, CREB and JNK. Data are represented as mean with SEM (*n* = 3), * *p* < 0.05, ** *p* < 0.01 *** *p* < 0.001.

Based on the in vitro data, an AD model was developed in mice to understand the Aβ-mediated neurodegeneration affecting the behaviour of the animals and also the inhibition of Wnt canonical signaling. An Aβ-induced mouse-AD model was created by injecting Aβ in the ventricular space in BALB/C mice brains using well reported protocols. To analyze the effect of Wnt inhibition in neurodegeneration in vivo, another model was created by injecting a proprietary Wnt antagonist AD201, a protein belonging to the secreted frizzled related protein (sFRP) family in a similar manner and compared with the well-characterized Aβ-AD model for AD-associated behavioural and pathological changes. The mice injected with Aβ showed loss of memory and were not able to locate the hidden platform in the water maze test. Significantly, similar results were observed in the mice injected with AD201. We also analyzed the changes in mice behaviour by calculating escape time and distance covered before being able to locate the hidden platform. On day 13 of the study, the mice belonging to the control group could find the platform within 35 s by covering the distance of 826.97 cm, while those injected with Aβ_1–42_ and AD201 took 41 s and 44.6 s covering the distance of 1181.39 cm and 1154.81 cm, respectively (Figure 6a(i,ii)). Furthermore, a clear loss of neurons in the cortex and hippocampal area in the Aβ-AD model and AD201-injected brain was observed in H and E staining of the brain sections (Figure 6b(i,ii)). Significantly, immunofluorescence and Congo red staining of Aβ aggregates in the brain sections indicated the presence of aggregated Aβ in both, Aβ and AD201 injected mice brain samples (Figure 6c(i,ii) respectively).

Gene expression study showed the expression of Nestin, Tuj1, ChAT and α7nAChR was lower than the control, whereas the expression of BACE1 was higher in the Aβ and AD201 injected mice, indicating the onset of neurodegeneration (Figure 6d(i,ii)). Wnt canonical and non-canonical pathway marker gene expression was similar to the in vitro AD model (Figure 6d(iii)). Dkk1 and GSK-3β was considerably higher than the control.

### 2.10. Downregulation of AD201 Significantly Restored the Expression of Neuronal Markers and Wnt Canonical Genes

**Figure 7:**
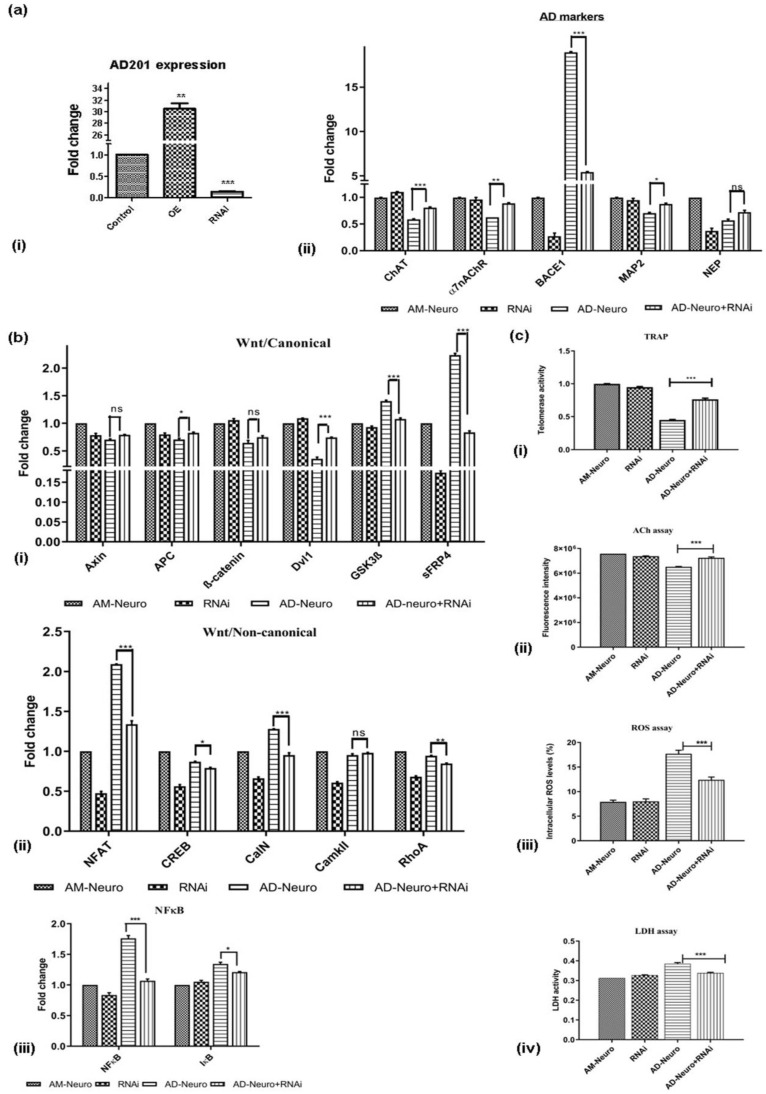
RNAi-mediated downregulation of Wnt antagonist, AD201 reverse neurodegeneration in in vitro AD model. (**a**) (**i**) qRT-PCR analysis showing AD201 expression in AD201-overexpressed (OE) and downregulated cells (RNAi), (**ii**) expression of AD-associated markers; ChAT, α7nAChR, BACE1, MAP2 and NEP by qRT-PCR analysis. (**b**) Gene expression analysis by qRT-PCR for (**i**) Wnt/canonical pathway markers: Axin, APC, ß-catenin, Dvl1, GSK3ß and sFRP4, (**ii**) Wnt/non-canonical pathway markers: NFAT, CREB, CalN, CaMKII and RhoA, (**iii**) NFκB and IκB. Data are represented as mean with SEM (*n* = 3), * *p* < 0.05, ** *p* < 0.01 *** *p* < 0.001. (**c**) Graphs showing (**i**) increase in telomerase activity, (**ii**) increase in ACh levels, (**iii**) decrease in intracellular ROS and (**iv**) decrease in LDH activity upon downregulation of AD201 in AD-Neuro cells. Data are represented as mean with SEM (*n* = 3), *** *p* < 0.001.

It was observed that exogenous AD201 treatment resulted in behavioural changes such as those associated with AD along with the loss of neuronal gene expression. Thus, it was hypothesized that inhibiting AD201 could lower the neuronal damage induced by Aβ via activation of Wnt-canonical signaling. Therefore, we downregulated the expression of AD201 using RNAi in AM-Neuro cells. Downregulation or silencing of AD201 was confirmed in AM-Neuro cells in comparison to the control and AD201 overexpressed cells (Figure 7a(i)). In line with our hypothesis, we observed that the neuronal damage following AD201 inhibition was significantly reduced in AD-Neuro cells, which was corroborated by a notable increase in the expression of functional cholinergic neuronal markers, such as ChAT and α7nAChR along with a lowered BACE1 expression (Figure 7a(ii)). Levels of MAP2 and NEP were significantly higher than that in the AD-Neuro cells. These results indicate that AD201 could have a role in promoting neurogenesis and could provide neuroprotection. Next, upon investigating the effect of AD201 inhibition on Wnt-related genes, we observed higher expression of Axin, APC, β-catenin and Dvl1, whereas there was a decrease in GSK-3β and sFRP4 expression in AD-Neuro cells with AD201 inhibition (Figure 7b(i)), indicating that Wnt-canonical signaling was expectedly activated upon downregulation of AD201. Moreover, expression of NFAT, CalN, CREB and RhoA was reduced whereas there was no significant change in CaMKII (Figure 7b(ii)). In addition, AD201 inhibition in AD-Neuro cells also inhibited activation of NFκB signaling (Figure 7b(iii)). Interestingly, there was also an increase in telomerase activity (Figure 7c(i)) suggesting a role of this enzyme in neuroprotection. These observations were further extended by neuronal functional analysis, wherein an increase in acetylcholine levels in AD201 cells was observed compared to AD-Neuro cells (Figure 7c(ii)). Finally, to study whether AD201 knockdown could indeed provide protection to neuronal insult, the effect of Aβ treatment in these cells was analyzed. Interestingly, and adding more evidence to the present hypothesis, AD201 inhibition in AD-Neuro cells was observed to reduce the Aβ-mediated oxidative stress (Figure 7c(iii)) and restored membrane integrity (Figure 7c(iv)).

## 3. Discussion

Several animal and cellular models of Alzheimer’s disease have been developed to establish the amyloid aggregation-based AD model [13,14,15,16]. Stem cells render a highly robust and malleable platform to understand neural development and also to unravel disease and its progression. Easy manipulation, both chemical and genetic, makes stem cells an excellent tool for disease modeling. Mesenchymal stem cells obtained from various sources are extensively utilized, owing to their accessibility, ease in handling and capacity to differentiate into various cell types [17,18,19]. MSC-derived functional neurons and glial cells are being utilized for replacement therapies in several neurological diseases [20]. In this study, we used human amniotic membrane–MSC-derived neuron as a robust cellular model for AD to investigate the underlying molecular mechanisms involved in Alzheimer’s disease. We obtained a population consisting maximally of neurons, fewer oligodendrocytes and astrocytes from AM-MSC using a novel differentiation cocktail. Differentiation of MSCs into neurons has been achieved previously by single or a combination of growth factors including bFGF, EGF and PDGF in addition to BDNF, β-nerve growth factor (NGF), glia-derived nerve factor (GDNF) and insulin-like growth factor-1 (IGF-1), which has been shown to promote neurogenesis and CNS development [21,22,23,24]. Previous study has also used cAMP signaling mediator, forskolin, with or without phosphodiesterase inhibitor such as dibutyryl-cAMP, which has shown to promote neuronal differentiation by activating MAPK [24]. Typically, the differentiation of MSCs achieved by these components not only takes a longer time span [25,26] but also often reverts to their original non-neuronal morphology. In our study, the differentiation cocktail containing a corticosteroid, a phosphodiesterase inhibitor and a nonsteroidal anti-inflammatory agent resulted in differentiation within 24 h, wherein the cells retain neuronal morphology and are robust and stable. The neuronal traits were further confirmed by the appearance of neurites and axonal processes, along with the expression of neuronal genes. Specifically, differentiation into cholinergic neurons was confirmed with ChAT and α7nAChR expression using the differentiation cocktail, further confirming the differentiation efficacy.

A complex balance between neurons and glial cells, such as astrocytes, oligodendrocytes and microglia, supports the normal functioning of neurons. Any damage to the neuron–glial association or dysregulation of cellular interactions often promotes AD-associated neurodegeneration. Insoluble Aβ deposition in the hippocampus and cortex has been reported to promote formation of neuritic senile plaques. Interaction of Aβ with neurons induces formation of Tau aggregates, triggers aberrant calcium influx and oxidative stress [27,28]. Influx of calcium along with excitatory neurotransmitter, glutamate leads to neuronal death [29]. Astrocytes which participate in synapse formation, brain homeostasis and the immune system are activated by Aβ. Insoluble Aβ aggregates activate glial cells which are in turn involved in its removal. Activation of astrocytes results in an increase in calcium influx and uptake of Aβ [30]. Astrocytes are also activated by cytokines released from microglia and these astrocytes further activate microglia and secrete ApoE2, which helps in the degradation of soluble Aβ. Microglia are often activated in response to damage to the brain and are involved in cytokines secretion and synaptic remodeling, thereby helping in Aβ clearance [29]. 

In the present study, on exposure to Aβ, we observed profound degeneration evident by loss of neuronal structure including loss of synapse and disappearance of neurites accompanied by the downregulation of neuronal markers. Selective loss of cholinergic neurons is observed in Alzheimer’s disease. Earlier reports have demonstrated significant reduction in ChAT gene expression and ChAT activity in AD patients [31,32]. In addition to ChAT, nicotinic acetylcholine receptors (nAChRs), especially α7 receptor (α7-nAChR), are also involved in cholinergic signaling and have altered function in AD [33]. We observed clear cholinergic deficits in the in vitro AD model exhibiting more pronounced AD-pathology. BACE1, a protease that cleaves the amyloid precursor protein at β-site which is further cleaved by γ-secretase to generate Aβ, is reported to show increased activity in brains of AD patients [19,34,35,36]. Increased BACE1 activity results in overproduction and accumulation of Aβ. BACE1 overexpression in the AD model observed in the present study, clearly corroborates previous findings that Neprilysin, an Aβ degrading enzyme, plays an important role in regulating the balance between Aβ production and degradation [3,37] and is reported to be downregulated in AD [38,39]. These findings indicate failure in the clearance of accumulated Aβ further deteriorating neuronal function [40]. The downregulation of neprilysin in our study provides an indication that the degradation of Aβ is affected in the AD model. 

Neuronal function is essentially dependent on the axonal transport of cargo which is mediated by microtubules that aid organelle transport and elongation of the axon. Inhibition of microtubules leads to interference in axonal transport. Microtubule-associated proteins, MAP2 and Tau (MAPT) are involved in microtubule stabilization and dynamics. MAP2 regulates neurite outgrowth and is abundant in CNS tissues. Previous findings show loss of MAP2 in animal models of AD [41]. Studies have shown that microtubule assembly is inhibited via degradation of MAP2 by Calpain which is activated by oxidative stress [42]. Unlike Tau, MAP2 does not form filaments upon hyperphosphorylation. Localization studies have revealed MAP2 in tangled neurons but not in neurofibrillary tangles [43]. A decline in MAP2 due to phosphorylation triggers neurotoxicity or synaptic toxicity as MAP2 is a dendritic protein [44]. Studies have reported elevated levels of neurotrophic factors such as FGF-2 in AD brain cause decrease in MAP2 [45]. MAPT gene mutation is associated with several neurodegenerative disorders. In AD, aberrant splicing of MAPT, hyperphosphorylation of Tau and aggregation into neurofibrillary tangles (NFTs) has been reported [46]. Our study reflected previous reports showing a decrease in MAP2 mRNA expression and MAPT protein in the AD model suggesting impaired microtubule dynamics and constrained axonal transport, eventually leading to apoptosis.

Protein phosphorylation regulates neuronal plasticity and neurotransmission via several signaling pathways. It further contributes to pathological conditions as it has an impact on memory and learning. AD has been linked to abnormal phosphorylation of the Tau protein [47]. Hyperphosphorylation of Tau leading to neurofibrillary tangle formation and synaptic dysfunction prior to deposition of Aβ are the hallmarks of AD [47]. Studies have shown that Tau interacts with numerous signaling proteins such as kinases and phosphatases including protein phosphatase 1 (PP1), protein phosphatase 2A (PP2A), glycogen synthase kinase 3β (GSK3β), cyclin-dependent protein kinase 5 (CDK5), src-family kinases (cSrc, Fgr, Fyn and Lck), growth factor receptor-bound protein 2 (Grb2), p85α and PLCγ [48,49,50,51]. GSK3ß, which destabilizes β-catenin, also phosphorylates Tau at approximately 36 residues, leading to its aggregation [52]. However, the main phosphorylation sites are Ser199, Thr231, Ser396 and Ser413. GSK-3β is activated in the early stage of Tau phosphorylation that decreases the binding of microtubules. Another protein, the cyclin-dependent protein kinase CDK5, can phosphorylate Tau at Thr231 [53] and Src family non-receptor tyrosine kinases (SFK) such as Fyn phosphorylates Tau at Tyr18 [54]. Studies have revealed that the interaction of Fyn with Tau causes a feedback loop and increase in enzymatic activity [55]. Tau targets Fyn in the postsynaptic space of dendrites where it affects synaptic plasticity [56,57,58]. 

As indicated previously, Aβ is a product of sequential cleavage of APP by β- and γ-secretase. γ-secretase is composed of four components: presenilins, nicastrin, anterior pharynx defective-1 (APH1) and presenilin enhancer 2 (Pen2). APH1 binds to nicastrin to form a complex which later interacts with PSEN and Pen2 to form an active γ-secretase complex. Studies have indicated that the expression of APH1-A significantly upregulated in patients with AD [59]. Overexpression of APH1-A in our study indicates that this gene product along with nicastrin and presenilins would be causing rise in γ-secretase activity further leading to increase in Aβ production. This is further confirmed by the presence of Aβ in the cell lysates as well as the supernatant.

Presenilins are catalytic subunits of the γ-secretase complex. Presenilin 2 or PSEN2 is associated with late age of onset and might lead to an increase in γ-secretase activity. It plays a role in plaque formation and is reported to be involved in the release of proinflammatory cytokines induced by Aβ [60]. Several studies have shown that PSEN2 levels are downregulated in AD [61]. Our study also showed a decrease in PSEN2 mRNA expression in Aβ treated cells.

Wnt signaling is highly regulated in embryonic development, neural development and homeostasis [62,63], and in the development of the central nervous system [64]. Previous findings have indicated that the canonical Wnt pathway is involved in the regulation of release of neurotransmitters, maintenance of long-term synaptic plasticity and transmission [63]. Thus, any alteration in the normal Wnt pathway regulation may lead to reduced synaptic function and contribute to the impaired condition. It is established that Wnt/β-catenin signaling is downregulated in aging and in familial AD [65,66]. GSK-3β is involved in hyperphosphorylation of Tau and in promoting the formation of neurofibrillary tangles (NFT) [67]. In concordance with this, in our study also, the AD model showed a clear elevation of GSK-3β and a decrease in expression of β-catenin. Several groups have previously reported reduced expression of β-catenin, higher GSK-3β and upregulated Wnt inhibitor, Dkk1 leading to neurodegeneration [68,69,70]. Importantly, we also showed for the first time, the overexpression of the Wnt antagonist, sFRP4. The decrease in β-catenin as seen in our study, could also be associated with the loss of α7nAChR expression since nicotine induces stabilization of β-catenin in Aβ induced toxicity [71]. Aberrant calcium homeostasis has been shown to trigger Aβ induced synapse and functional loss of neurons [72,73]. Similarly, an increase in the indicators of the Wnt-calcium pathway, that is, CalN and NFAT was observed in the present study. This is in agreement with previous reports indicating an activation of CalN and dephosphorylation of NFAT in AD brains [74,75]. Activation of CalN also has been linked to hyperphosphorylation of Tau [76], and active NFAT and CalN was previously shown to induce morphological changes including reduction in spine density and dendritic simplification [77]. A reduced expression of CREB and CaMKII observed in our study is indicative of inhibition of neurite growth and compromised synaptic plasticity as reported previously for AD wherein downregulation of CREB inhibited neurite growth [78] and dysregulation of CAMKII altered synaptic plasticity [79].

In the context of diseases, one of the major drivers of tumorigenesis is an upregulated Wnt pathway. However, during the onset of neurodegenerative diseases such as AD, Wnt downregulation and suppression is a primary causative factor. Thus, there is a paradoxical situation with regard to representation of Wnt in cancer and neurodegeneration. Although the molecular machinery involved in maintaining neural function in neurodegenerative diseases may be shared with oncogenic pathways, susceptibility to one may provide protection against the other disease. Abnormal Wnt signaling is associated with several diseases including cancer and neurodegenerative diseases like AD [80]. In our earlier studies, we have reported that inhibition of Wnt signaling using Wnt antagonist leads to tumor suppression [81]. Some of the major Wnt factors are reported to have differential expression in cancers and AD. For instance, higher expression of GSK-3β is observed in colon, liver, ovarian, and pancreatic cancers [82] whereas overexpression of GSK-3β promotes hyperphorphorylation of Tau in AD [83]. Dvl1 that is downregulated in AD, is overexpressed in several cancers including cervical cancer, non-small cell lung cancer, mesothelioma, breast and prostate cancer [84,85,86]. A recent study by Dou et al. confirmed that levels of APC and Axin are upregulated in β-catenin mutated tumors such as endometrial carcinoma [87], which was observed to be downregulated in AD in the present study.

Maintenance of telomere length is associated with senescence and the enzyme telomerase reverse transcriptase (hTERT) maintains the telomeric length in the dividing cells. Apart from cellular aging, environmental factors and lifestyles can have an impact on telomere length [88,89,90]. Reports have suggested that age and weight are negatively correlated with hTERT gene expression [91]. Oxidative stress and inflammation are known to have a major impact on telomere activity [89,92]. Shortening of telomere length serves as a cellular mechanism for accumulation of DNA damage due to oxidative stress. Further, oxidative stress inhibits telomerase and impedes the recognition by telomere-binding proteins leading to telomere uncapping and shortening [93]. Interestingly, and to further confirm a successful model creation of AD derived from MSCs, we observed a significant reduction in both hTERT expression and in telomerase activity in AD-Neuro cells. It has been reported that during cellular stress in AD, TERT is exported from the nucleus and translocated to mitochondria to improve its functioning and decrease ROS [94,95]. Loss of TERT in our study could indicate reduction in resistance against ROS and decline in mitochondrial function. 

Acetylcholine (ACh), a key neurotransmitter, is often reduced in AD along with a decrease in the ACh inactivating enzyme, acetylcholine esterase (AChE) [96,97,98]. Some of the most commonly used drugs for AD, such as rivastigmine and donepezil, act by suppressing AChE and restoring ACh function. In order to analyze the robustness of the AD-Neuro model, we used it to analyze the effect of AD drugs, rivastigmine, donepezil and memantine wherein we could see a clear increase in ACh levels, expression of neuronal markers and cholinergic markers ChAT, α7nAChR, MAP2 and NEP. These results reveal that there is a significant rescue of molecular markers for neurodegeneration upon drug AD treatment. In addition to the functional and cholinergic markers, we also observed a decrease in BACE which possibly facilitated a reduction in Aβ accumulation. Mechanistically, we could show the reduction in Wnt inhibitors as seen by a decrease in GSK-3β and sFRP4, suggesting an activation of the Wnt/β-catenin pathway.

Oxidative stress arises due to an imbalance between ROS generation and its scavenging brought about by antioxidative defence of the cells. In AD, a study performed at different stages of the disease showed a rise in lipid peroxidation [99,100], protein oxidation [101] and damage of nucleic acids [102,103]. Although the cause–effect relationship between Aβ and oxidative stress is still unknown, oxidative stress is considered as one of the pathological elements in AD. Similarly, in the present study intracellular ROS activity was found to be higher, which upon drug treatment was lowered indicating the decrease in oxidative stress induced by Aβ treatment. Previously, Goschorska M. et al. reported the antioxidant activity of rivastigmine and donepezil in THP-1 cells [104]. The increase in LDH in the AD-Neuro cells correlates well with studies showing aging-related mitochondrial dysfunction in AD, wherein higher levels of lactate have been observed in AD brains [105]. The increase in LDH activity indicates the conversion of pyruvate to lactate suggesting altered metabolism and neuronal damage. The efficacy of the AD-neuro model was further confirmed in our study wherein drug treatment significantly lowered the LDH upsurge.

As we could see a remarkable presence of Wnt antagonists in the in vitro AD model, we wanted to compare the effect of a proprietary Wnt antagonist, AD201, with the traditional Aβ treatment in a mouse model to see if AD201 could induce AD-like neurodegeneration. The observation of AD-like traits both with behaviour patterns of memory deficits, loss of neurons in cortex and hippocampal area, amyloid plaques and mechanistically in terms of upregulation of GSK-3β and Dkk1 similar to the classic Aβ induced AD model, gives a promising lead to the effect of Wnt antagonism in AD-like neurodegeneration. These observations are in concordance with the recent reports in J20-APP transgenic and wild-type mice by Tapia-Rojas and Inestrosa [106], wherein they demonstrate that Wnt inhibitors accelerates the appearance of neuropathological hallmarks of AD 

Based on these observations, we investigated the effect of RNAi-mediated inhibition of AD201 in the AD-Neuro in vitro model. We could observe an emphatic reversal of neurodegeneration, restoration of neuronal function, enhancement of mitochondrial function and reduction in oxidative stress, in addition to enhanced telomerase activity and activation of the Wnt mediators. These findings could be the forerunner for the development of inhibitors of AD201 to help in the reversal of neurodegeneration as reviewed recently by Jia et al. [66]. RNAi-mediated silencing of the Wnt antagonist could thus activate Wnt signaling and most likely rescue the degenerating neurons as well as promote neuron survival.

## 4. Methods

### 4.1. Isolation of MSC from Amniotic Membrane

Term placentas from pregnancies were collected from Omega Multispeciality Hospital, Bangalore, India. The amniotic membrane was peeled and washed thrice in phosphate-buffered saline (PBS; Sigma-Aldrich, St Louis, MO, USA) and 100 U/mL Penicillin-Streptomycin (Gibco, Grand Island, NY, USA). The amniotic membrane–MSCs (AM-MSCs) were isolated as previously reported by Warrier S. et al. [12]. The cells were cultured in Dulbecco’s Modified Eagle Medium, high glucose (DMEM; Gibco, MA, USA) containing 10% fetal bovine serum (FBS; Gibco, Grand Island, NY, USA) at 37 °C and 5% CO_2_. AM-MSC at passage 2 were characterized as per standards established by the International Society for Cellular Therapy (ISCT) for defining human MSC [107] by molecular analysis, CFU-F assay, immunofluorescence, flow cytometry and trilineage differentiation. Detailed methods are described in the Supplementary Methods.

### 4.2. Induction of Neurogenesis in AM-MSC

AM-MSC at passage 3 were induced for differentiation into neuronal cells using a cocktail of proprietary compounds DC304 (1 µM, an anti-inflammatory corticosteroid), DC305 (0.5 mM, a phosphodiesterase inhibitor) and DC306 (100 µM, a non-steroidal anti-inflammatory agent) in DMEM-HG. Differentiated cells showing neuronal morphology were termed as AM-Neuro.

### 4.3. Cell Viability and Mitochondrial Staining

AM-Neuro cells were stained with 1 µM Calcein-AM (BD biosciences, San Diego, CA, USA) for determination of cell viability for 30 min. Tetramethyl rhodamine (TMRE, Invitrogen, Carlsbad, CA, USA) staining was conducted with 0.3 µM TMRE dissolved in methanol to observe active mitochondria. After staining for both assays, cells were observed using a Nikon TE-2000U microscope.

### 4.4. Molecular Analysis of Differentiation

Expression of neuronal markers was determined in AM-Neuro by qRT-PCR using primers indicated in Table S1. mRNA expression of the genes was normalized to the housekeeping gene, GAPDH and the results were calculated based on the 2^−ΔΔCT^ method and expressed as fold change [108]. 

### 4.5. Immunofluorescence and Flow Cytometry

Immunofluorescence and flow cytometry was performed for the presence of neuronal markers in AM-Neuro cells by following the procedure mentioned in the Supplementary Methods and antibodies in Table S2. 

### 4.6. Induction of Neurodegeneration

Synthetic Aβ_1–42_ (Sigma Aldrich, St Louis, MO, USA) stocks were prepared following the method described by Arrazola, M. et al., 2017 [109]. AM-Neuro cells were treated with 2.5 µM Aβ_1–42_ oligomers for 24 h at 37 °C to induce degeneration. These cells were termed as AD-Neuro. Onset of neurodegeneration was assessed by morphological examination, qRT-PCR, immunofluorescence and cytotoxicity assays- ROS and LDH and acetylcholine assay. Wnt signaling marker expression was also determined in AD-Neuro cells by PCR. All the primer sequences are indicated in Table S1. Details on methods for these assays are mentioned in the Supplementary Methods.

### 4.7. TRAP Assay

Variations in telomerase are associated with several neurological disorders and are determined by Telomerase Repeated Amplification Protocol (TRAP) as mentioned in the Supplementary Methods. 

### 4.8. Drug Studies

Rivastigmine tartrate (R), donepezil hydrochloride (D) and memantine hydrochloride (M) were procured from Sigma Aldrich, St Louis, MO, USA and the stocks were prepared [110,111,112,113]. Rivastigmine, donepezil and memantine were used at 2 µM, 4 µM and 3 µM, respectively, for the subsequent experiments. 

### 4.9. Animal Study

The study involved 3.5–4 month-old male BALB/c mice weighing 25–35 g, which were grouped based on the treatment conditions. The water maze task was performed to measure long-term spatial learning and memory function, as described previously [114]. Training was conducted over 4 consecutive days, with 2 trials per day. During the first two days, mice were trained to find a dark-coloured cylindrical platform with a diameter of 10 cm, sitting 0.5 cm above the water surface. On the 3rd and 4th day, the platform was moved to the opposite quadrant and submerged 1 cm below the surface of the water. The escape latency, swimming distance and swimming speed were analyzed. AD-specific neurodegeneration was induced in the mice with intracerebroventricular injection of 25 µM Aβ_1–42_ and one group was injected with 1 µg AD201 (R&D systems). The animals were allowed to rest and observed for 8 days. The animals were allowed to perform the same task for two consecutive days. Further, sectioning and tissue preparation, immunohistochemistry and molecular analysis was carried out on the brain samples. Additionally, Congo red staining was performed as previously reported [115,116]. Detailed procedures are mentioned in the Supplementary Methods.

### 4.10. AD201 Silencing Study

The effect of the Wnt antagonist, AD201 knockdown, was examined in AM-Neuro and AD-Neuro cells. AD201 dsRNA was synthesized using MEGAscript RNAi Kit (Ambion, Life Technologies, CA, USA, cat. no. AM1626) as per manufacturer’s instructions. AM-Neuro cells were transfected with 1 µg AD201 dsRNA using Lipofectamine 3000 (Invitrogen, CA, USA) for 24 h. AD201 silencing was confirmed by gene expression. 

### 4.11. Statistical Analysis

All the represented data are the mean and standard error mean (SEM) obtained from three independent experiments performed in triplicate. Student’s *t*-test and one-way analysis of variance (ANOVA) followed by Tukey’s post-hoc test was performed to derive statistical significance. *p* < 0.05 was considered significant for all statistical analyses compared to the respective control.

## 5. Conclusions

The present study demonstrated a robust in vitro model of AD derived from stem cells which efficiently respond to AD drug treatment. Thus, the derived AD platform not only can facilitate drug efficacy studies but can also be used as a model for understanding the complex molecular mechanisms involved in the onset of the disease. Using this approach, we obtained an initial lead that a proprietary Wnt antagonist, AD201, could be a reliable indicator of the disease and inhibition of which significantly ameliorated the molecular markers of AD. This opens up a tantalizing possibility for the development of drug targets suppressing AD201 and as an extension, the development of AD201 as a novel early biomarker for Alzheimer’s disease.

## Data Availability

All the data generated or analyzed during this study are included in this article.

## Supplementary Materials

The following supporting information can be downloaded at: https://www.mdpi.com/article/10.3390/bioengineering10020192/s1, Table S1: List of primers used in the in vitro and in vivo study of AD. Table S2: List of primary and secondary antibodies used in the study. Data S1: Compilation of agarose gel images. corresponding to Figure 1 (S1-a), Figure 2 (S1-b) and Figure 4 (S1-c). Data S2: Compilation of Western blots corresponding to Figure 3.

## List of Abbreviations

ACh, acetylcholine; AChE, acetylcholinesterase; AD, Alzheimer’s disease; AM-MSC, amniotic membrane-mesenchymal stem cells; APP, amyloid precursor protein; Aβ, amyloid-beta; BACE1, β-APP cleaving enzyme; bp, base pair; BSA, bovine serum albumin; CalN, calcineurin; cDNA, complementary DNA; CFU-F, colony formation units-fibroblasts; Cx-Cortex; D, donepezil hydrochloride; DAPI, 4′,6-diamidino-2-phenylindole dihydrochloride; DMEM-HG, Dulbecco’s Modified Eagle Medium, high glucose; dsRNA, double stranded RNA; FBS, fetal bovine serum; H and E, Haematoxylin and Eosin staining; Hpc, hippocampus; hTERT, human telomerase reverse transcriptase; ITS, insulin transferrin selenium; LDH, lactate dehydrogenase; M, memantine hydrochloride; NFAT, nuclear factor of activated T cells; PBS, phosphate-buffered saline; PFA, paraformaldehyde; qRT-PCR, quantitative-reverse transcriptase polymerase chain reaction; R, rivastigmine tartrate; RNAi, RNA interference; ROS, Reactive oxygen species; RT-PCR, reverse transcriptase polymerase chain reaction; sFRP, secreted frizzled-related protein; TMRE, tetramethylrhodamine ethyl ester, perchlorate; TRAP, telomerase repeated amplification protocol; WHO, World Health Organization.
