# Supplementary material for: Identification of a Novel Wnt Antagonist Based Therapeutic and Diagnostic Target for Alzheimer’s Disease Using a Stem Cell-Derived Model
Source: Bioengineering (Basel). 2023 Feb 2;10(2):192. doi: 10.3390/bioengineering10020192 (PMC9952699; doi:10.3390/bioengineering10020192)
Supplement: Supplementary file 1 [file bioengineering-10-00192-s001.zip › AMAD supplementary 231022.pdf]

Supplementary information

Identification of a novel Wnt antagonist based therapeutic and diagnostic target for Alzheimer's disease using a stem cell derived model

Supplementary methods

Molecular analysis

Total RNA was extracted from the cells using RNAiso plus reagent (Trizol, TaKaRa, Japan) and complementary DNA (cDNA) synthesis was carried out using PrimeScript™ 1st strand cDNA synthesis kit (TaKaRa, Japan) and gene expression was analysed by PCR using 2X EmeraldAmp® GT PCR Master Mix (TaKaRa, Japan) with respective primers (procured from Sigma, sequence as indicated in Table S1) and resolving the amplified products on 2% agarose gel.

Real-time PCR or qRT-PCR was performed using SYBR® Premix Ex Taq™ (TaKaRa, Japan) in MicroAmp Optical 8-tube and cap strips (Applied Biosystems, USA) with PCR parameters were as per manufacturer's instructions. Primer sequences are indicated in Table S1.

CFU-F assay

AM-MSC at passage1 and 2 were plated at $1X10^4$ cells per 60mm culture dish and incubated for 7 days at 37°C in 5% humidified CO_2. The cells were washed with PBS and stained with 0.5% crystal violet (HiMedia, India). Isolated colonies with more than 50 cells were counted and the colony formation efficiency was estimated (number of colonies per 10^4 cells).

Immunofluorescence

Spent medium was removed from the cells and a PBS wash was given. The cells were fixed with 4% paraformaldehyde (PFA) for 15min and permeabilised with 0.1% Triton X-100 in PBS for 15min at room temperature. The cells were washed with PBS and then blocked with 3% bovine serum albumin (BSA) at room temperature for 30min. The cells were then incubated

with specific primary antibody at 4°C, overnight (Table S2). The cells were washed once with PBS with 0.1% tween-20 (PBST) and incubated with respective secondary antibody at room temperature for 1h (Table S2). After removing the secondary antibody, the cells were washed with PBST and then the nucleus was stained with 4',6-diamidoino-2-phenylindole (DAPI) (HiMedia, India, cat#MB097, 1:10000 dilution). The cells were observed on an inverted fluorescent microscope Nikon Eclipse TE 2000-U (Nikon, Japan) and Olympus IX73 (Olympus, Japan). They were imaged using Qimaging-QICAM-fast 1394 software and further analysed with ImageJ.

Flow cytometric analysis

Cells were fixed with 4% PFA at room temperature for 20min followed by PBST wash. The cells were then either permeabilized with 0.1% Triton X-100 in PBS or directly blocked with 3%BSA for 30min and then incubated with specific antibodies at room temperature for 2h (Table S2). The cells were either incubated with secondary antibody (Table S2) or directly acquired using BD-FACS Calibur flow cytometer (BD Biosciences, USA) along with respective isotype controls and data was analyzed by Cell Quest Pro Software.

Trilineage differentiation in AM-MSC

AM-MSC were characterized for differentiation into adipogenic, osteogenic and chondrogenic lineages.

Adipogenesis was induced using medium containing dexamethasone (1μM), isobutyl methyl xanthin (0.5mM), indomethacin (200μM) and insulin (1μg/mL) in DMEM-HG for about 15-20days till the lipid droplets were visible which were stained by Oil red 'O' after fixation.

AM-MSC were induced for osteogenic differentiation by using medium containing ascorbic acid (500μg/mL), β-glycerophosphate (10mM), dexamethasone (1μM) in DMEM-HG for 20 days. The calcium deposits were stained with 2% Alizarin Red to confirm differentiation.

Chondrogenic differentiation was achieved using medium containing ascorbic acid (50µg/mL), dexamethasone (0.1µM), insulin transferrin selenium (ITS) (1X) transforming growth factor (TGF-β1) (10ng/mL) and sodium pyruvate (100µg/mL) for 20 days. The differentiated cells were fixed and stained with 1% Alcian blue to stain the glycosaminoglycans.

All the differentiation components were purchased from Sigma-Aldrich, St Louis, MO, USA. The stained cells were observed under bright-field phase contrast microscope (Nikon-Eclipse TE2000-S; Japan) and the images were captured using QCapture software.

ROS assay

Generation of reactive oxygen species (ROS) taking place during the process of degeneration was analysed using Fluorometric intracellular ROS kit (#MAK142, Sigma Aldrich, USA). Cells were seeded in triplicates at density of $1X10^4$ cells per well of 96-well plate. Next day, $A\beta_{1-42}$ was added for 24h followed by ROS estimation by detecting fluorescence at λex-650/ λem-675 nm using EnSight™ Multimode plate reader (Perkin Elmer, USA).

LDH assay

Cellular damage and lactate deficit was assessed by determining the lactate dehydrogenase (LDH) activity using Pierce LDH cytotoxicity assay kit (cat# 88953, Thermo Fisher Scientific, USA) by measuring absorbance at 490nm and 680nm.

Acetylcholine activity

Acetylcholine deficiency is linked to pathogenesis of AD, therefore, we determined Acetylcholine levels in the control and treatment samples using Amplex Red Acetylcholine/Acetylcholinesterase assay kit (Cat# A12217, Molecular Probes, USA) by following manufacturer's instructions.

Western Blotting

Cells were lysed using RIPA buffer containing protease inhibitors and the resulting suspension was centrifuged at 12000rpm for 15min. the clear cell lysates were transferred to clean tube

and stored at -80°C till further use. The protein samples were then heated with Laemmli buffer and loaded on 12% SDS-Polyacrylamide gel. After resolving the samples, the proteins were transferred onto PVDF membrane using semi-dry blotting apparatus. The membrane was blocked with 3% BSA in 1X TBST to avoid non-specific binding. The blots were then incubated with primary antibodies at 1:1000 concentration on a rocking platform at 4°C overnight. The blots were then washed thrice with 1X TBST and HRP conjugated secondary antibody at 1:2000 diltution was added and incubated for 2h at room temperature. The blots were again washed thrice with 1X TBST and developed using Westernbright ECL HRP substrate (Advansta, USA) on ChemiDoc™ XRS+ (Bio-Rad, USA). Densitometric analysis was performed for the blots using ImageJ software.

ELISA

Homogenates used for Western analysis were diluted in 1:5 ratio and Amyloid β levels were measured by indirect ELISA. Also, the supernatant/media from the cells was used to estimate the secreted Amyloid β levels. The cell lysates and supernatant were incubated overnight on a coated 96-well plate in triplicates at 4°C. The wells were washed thrice with 1XTBST and polyclonal anti-human Amyloid β antibody was added and the plate was placed on rocker for 4h. the primary antibody was collected and the wells were again washed thrice with 1XTBST and HRP conjugated secondary antibody was added and incubated for 2h at room temperature. After washing, substrate-TMB/H2O2 was added and incubated for 30min at room temperature in dark. The reaction was stopped by adding 1M sulphuric acid and the absorbance was recorded at 450nm using EnSight™ Multimode plate reader (Perkin Elmer, USA).

TRAP

100µL freshly prepared TRAP (NP40 lysis buffer) buffer was added to the cell pellet and incubated on ice for 30min. The suspension was centrifuged at 4°C for 30min at high speed.

After protein estimation by Bradford method, a PCR reaction was set as follows- 500ng protein lysate, 10µL SYBr® Premix Ex Taq II™ (TaKaRa, Japan), 1µL ACX primer and 1µL TS primer; final volume made up to 20µL with nuclease-free water. The reaction was set as 95ºC-30s, 60ºC- 30s and 72ºC-1min for 32 cycles. Telomerase activity in samples was calculated based on the threshold cycle value (Ct).

Section and tissue preparation

Mice were euthanized by trained personnel using standard procedure and the brains were dissected from the body and fixed with 4% PFA overnight at 4°C. The brain tissue was dissected in mid-sagittal plane and embedded in paraffin. 50µm-thick coronal sectioning and H&E staining was performed at Anand Diagnostics, Bangalore, India.

Immunohistochemistry on mouse brain tissue

Immunohistochemical staining was done by deparaffinizing brain sections and antigen retrieval. Slides were incubated with Rabbit anti-Human Amyloid- β antibody followed by secondary antibody (Table S1) and nucleus was stained with DAPI.

Congo red

Congo red staining was performed on the unstained sections for detection of amyloid peptides. The sections were deparaffinised with xylene and washed in distilled water for 30s. The slides were then incubated for 20min in alkaline saturated NaCl solution made in 80% ethanol followed by incubation in alkaline Congo red solution made in NaCl saturated 80% ethanol. The slides were rinsed with 4 dips each of 95% and 100% ethanol. The slides were then mounted and allowed to air dry. The slides were then observed using phase contrast microscope (Nikon eclipse TE 2000-S) and the images were captured using Q-capture software.

Molecular analysis of mouse brain

Mice were euthanized by standard procedure at CLATR by trained personnel and the brain tissue was washed in PBS. The cerebral cortex and hippocampus were dissected out. RNA was

extracted using RNAiso plus reagent and cDNA was prepared as previously mentioned. Gene expression analysis was carried out using specific mouse primers (Table S1).

List of abbreviations

bp, base pair; BSA, Bovine serum albumin; cDNA, complementary DNA; Cx-Cortex; DAPI, 4′,6-diamidino-2-phenylindole dihydrochloride; H&E, Haematoxylin and Eosin staining; Hpc-Hippocampus; ITS, Insulin transferrin selenium; PBS, Phosphate-buffered saline; PFA, Paraformaldehyde; RNAi, RNA interference

Supplementary tables

Table S1: List of primers used in the *in vitro* and *in vivo* study of AD

Gene	Sequence	Product length (bp)	Annealing temperature (ºC)
Human primers			
GAPDH	F: 5'-CGACCACTTGTCAAGCTCA-3'<br>R: 5'-AGGGGAGATTCAGTGTGGT-3'	202	59
vimentin	F: 5'-GGTACAAGTCCAAGTTTGCTGACCT-3'<br>R: 5'-CATTGAGCAGCTCTTGGTATTCACG-3'	297	60
CD90	F: 5'-ATGAACCTGGCCATCAGCA-3'<br>R: 5'-GTGTGCTCAGGCACCCC-3'	218	56
CD73	F: 5'-GCCGCTTTAGAGAATGCAAC-3'<br>R: 5'-CTCGACACTTGGTGCAAAGA-3'	234	55
CD105	F: 5'-CCACTAGCCAGGTCTCGAAG-3'<br>R: 5'-GATGCAGGAAGACACTGCTG-3'	192	53
CD34	F: 5'-CTACAACACCTAGTACCCTTGGA -3'<br>R: 5'-GGTGAACACTGTGCTGATTACA-3'	185	54
synapsin1	F: 5′-TCAGACCTTCTACCCCAATCA-3′	127	55

	R: 5′-GTCCTGGAAGTCATGCTGGT-3′		
tuj1	F: 5’-CGGGGCCGCGGCTATAAGAG-3’<br>R: 5’-CAGCCTCACCTTGGCCCCGA-3’	168	60
neurofilament	F: 5’-CGCTATGCAGGACACGATCA-3’<br>R: 5’-CTGGTCTGTAAACCGCCGTA-3’	255	60
neuropilin	F: 5’- GAAGGCAACAACAACTATGA-3’<br>R: 5’- ATGCTCCCAGTGGCAGAATG-3’	354	60
ChAT	F: 5’-ACTGGGTGTCTGAGTACTGG-3'<br>R: 5'-TTGGAAGCCATTTTGACTAT-3'	451	53
α7nAChR	F: 5’-CCGACTCTGGGGTAGTGTGT-3’<br>R: 5’-ATGGTGCAGATGATGGTGAA-3’	250	55
TH	F: 5’-GCGCAGGAAGCTGATTGCTG-3’<br>R: 5’-TGTCTTCCCGGTAGCCGCTG-3’	200	56.8
BACE1	F: 5’- ACCGACGAAGAGTCGGAGGAG-3'<br>R: 5’-CACAATGCTCTTGTCATAG -3’	651	55
MAP2	F: 5′-TGCCATCTTGGTGCCGA-3’<br>R: 5′-CTTGACATTACCACCTCCAGGT-3’	360	55
Neprilysin	F: 5’-GCCTCAGCCGAACCTACAAG-3’<br>R: 5’-AATTTGCACAACGTCCTCAAGTT-3’	96	54
β-catenin	F: 5’-CGTCCACAACACTCTGGCTA-3’<br>R: 5’-GCCAGCACTTCACTGCAATA-3’	159	55

GSK3- β	F: 5’-ACTCCAGTGGCGAGAAGAAA-3’<br>R: 5’-TTGAGGACAGCAGTGTCAGG-3’	241	55
sFRP4	F: 5’-CGATCGGTGCAAGTGTAAAA-3’<br>R: 5’-GACTTGAGTTCGAGGGATGG-3’	181	60
Dkk1	F: 5’-TCCGAGGAGAAATTGAGGAA-3’<br>R: 5’-CCTGAGGCACAGTCTGATGA-3’	157	52
NFκB	F: 5’-ATGGCTTCTATGAGGCTGAG-3’<br>R: 5’-GTTGTTGTTGGTCTGGATGC-3’	128	62
IKB	F: 5’-TTGCTGAGTGACATTGCCTC-3’<br>R: 5’-GAAGCTCCAGTAGTCGACGG-3’	252	54
Axin	F: 5’-CTGGCTATGTCTTTGCACCA-3’<br>R: 5’-AGGAGGGATTCCATCTACGC-3’	113	58
APC	F: 5’-CCAGGAACTTCTTCAAAGCG-3’<br>R: 5’-TATGGGCAGCAGAGCTTCTT-3’	181	53
Dvl1	F: 5’- CCACCCTGAACCTCAACAGT-3’<br>R: 5’-CCTTCACTCTGCTGACTCCC-3’	202	54
NFAT	F: 5’-TTCGGAAAGGAGAGACGGAC-3’<br>R: 5’-ACAGGACCATCTTCTTCCCG-3’	213	55
CREB	F: 5’-GCTGGCTAACAATGGTACCG-3’<br>R: 5’-CTGGGGAGGATGCCATAACA-3’	242	55
CalN	F: 5’-GATGATGGTGGGGAACAATC-3’<br>R: 5’-GCCACCTACAACAGCACAGA-3’	127	54
CaMKII	F: 5’-CCGACGACTACCAGCTCTTC-3’<br>R: 5’-TCCGACCCTCACGTTCTAGT-3’	159	55

RhoA	F: 5'-CCATCGACAGCCCTGATAGT-3'<br>R: 5'-CGCCAATCCTGTTTGCCATA-3'	204	58
hTERT	F: 5'-CGTGGTTTCTGTGTGGTGTC-3'<br>R: 5'-CCTTGTCGCCTGAGGAGTAG-3'	214	60
TRAP	ACX: 5'-GCGCGGCTTACCCTTACCCTTACCCTAACC-3'<br>TS: 5'-AATCCGTCGAGCAGAGTT-3'	--	60
AD201 RNAi T7 promoter	F: 5'-TAATACGACTCACTATAGGGAGACCATTTGCACCCTGGAGTTC-3'<br>R:3'-TAATACGACTCACTATAGGGAGATCCACTTAACATCCTCCGGG-3'	210	60
Presenilin2	F:5'- ACGACCCGGAGATGGAAGAAG-3'<br>F:5'- CAGCGTGGTATTCCAGTCCC-3'	212	60
APH1-A	F:5'-CATTTTTCTGGCTGGTCTC-3'<br>R:5'-AACCCCTCATCTGCCTTCTT-3'	188	60
Mouse Primers			
GAPDH	F: 5'-TGTGAACGGATTTGGCCGTA-3'<br>R: 5'-ACTGTGCCGTTGAATTTGCC-3'	159	55
nestin	F-5'-CAGCGTTGGAACAGAGGTTGG-3'	389	60

	R-5'-<br>TGGCACAGGTGTCTCAAGGGTAG-3'		
tuj1	F: 5'-TCCAGGAGCTGTTCAAGCG-3'<br>R: 5'-TCGGACACCAGGTCGTTC-3'	142	60
ChAT	F: 5'-CCCTGCCAGTCAACTCTAGC-3'<br>R: 5'-ATACAGAGAGGCTGCCCTGA-3'	184	55
α7nAChR	F: 5'-CCGACTCTGGGGTAGTGTGT-3'<br>R: 5'-ATGGTGCAGATGATGGTGAA-3'	250	54
BACE1	F: 5'-ATGTGGAGATGACCGTAGGC-3'<br>R: 5'-TACACACCCTTTCGGAGGTC-3'	160	55
β-catenin	F: 5'-CGTCCACAACACTCTGGCTA-3'<br>R: 5'-GCCAGCACTTCACTGCAATA-3'	159	55
Dkk1	F: 5'-TCCGAGGAGAAATTGAGGAA-3'<br>R: 5'-CCTGAGGCACAGTCTGATGA-3'	163	52
GSK3- β	F: 5'-ACTCCAGTGGCGAGAAGAAA-3'<br>R: 5'-TTGAGGACAGCAGTGTCAGG-3'	241	55
Axin	F: 5'-CTGGCTATGTCTTTGCACCA-3'<br>R: 5'-AGGAGGGATTCCATCTACGC-3'	113	58
CalN	F: 5'-GATGATGGTGGGGAACAATC-3'<br>R: 5'-GCCACCTACAACAGCACAGA-3'	127	54
NFAT	F: 5'-TTCGGAAAGGAGAGACGGAC-3'<br>R: 5'-ACAGGACCATCTTCTTCCCG-3'	213	55
CREB	F: 5'-GCTGGCTAACAATGGTACCG-3'<br>R: 5'-CTGGGGAGGATGCCATAACA-3'	242	55

JNK	F: 5’-AGAAGCTCCACCACCAAAGA-3’	154	55
	R: 5’-CTGTGCTAAAGGCGAGGGCT-3’		

Table S2: List of primary and secondary antibodies used in the study

Antibody	Company	Cat#, RRID	Dilution Immunofluorescence (IF)/ Flow cytometry (F)
Primary antibodies			
PE Mouse anti-Human CD73	BD Pharmingen™, USA	561014; RRID: AB_2033967	IF 1:500
Alexa fluor 488 Mouse anti-Human Vimentin		562338, RRID: AB_10896994	IF 1:500
PE Mouse anti-Human CD34		clone 563, 550761, RRID: AB_393871	IF 1:500, F 1:200
FITC Mouse anti-Human CD45		555482, RRID: AB_395874	IF 1:500
FITC recombinant anti-Human CD90	Miltenyi Biotec, CA	clone REA897, RRID: AB_2726842	F 1:250
PE recombinant anti-Human CD105		clone REA794, RRID: AB_2654427	F 1:250
Rabbit anti-Human Amyloid beta	Thermo Fisher Scientific, USA	71-5800, RRID: AB_2533989	IF 1:1000
Rabbit anti-Synapsin1	Invitrogen, USA	A6442, RRID: AB_2536207	IF 1:1000, F 1:1000
Mouse anti-beta tubulin (tuj1)	Thermo Fisher Scientific, USA	480011, RRID: AB_2532242	IF 1:1000

Rabbit anti-Human MAPT	Cloud-Clone corp, USA	PAB983Hu01	IF 1:500
Rabbit anti-Human ChAT		PAB929Hu01	IF 1:500, F 1:200
Mouse Anti-human Beta Actin	Invitrogen	MA1-140, clone 15G5A11/E2 RRID: AB_2536844	WB 1:1000
Secondary antibodies			
Goat anti-Rabbit Alexa Flour 488 antibody	Thermo Fisher Scientific, USA	A32731, RRID: AB_2633280	1:2000
Goat anti-Mouse Alexa Fluor 488 antibody		A11029, RRID: AB_2534088	
Goat anti-Rabbit Alexa Fluor 594		11037, RRID: AB_2534095	
Goat Anti-Rabbit HRP		32460, RRID: AB_1185567	
Goat Anti-Mouse HRP		32430, RRID: AB_1185566	
