# Supplementary material for: Identification of a Novel Wnt Antagonist Based Therapeutic and Diagnostic Target for Alzheimer’s Disease Using a Stem Cell-Derived Model
Source: Bioengineering (Basel). 2023 Feb 2;10(2):192. doi: 10.3390/bioengineering10020192 (PMC9952699; doi:10.3390/bioengineering10020192)
Supplement: Supplementary file 1 [file bioengineering-10-00192-s001.zip › Supplementary Data 231022.pdf]

Data S1. Compilation of agarose gel images (a-c). Agarose gel image corresponding to Figure 1 (S1-a), Figure 2 (S1-b) and Figure 4 (S1-c).

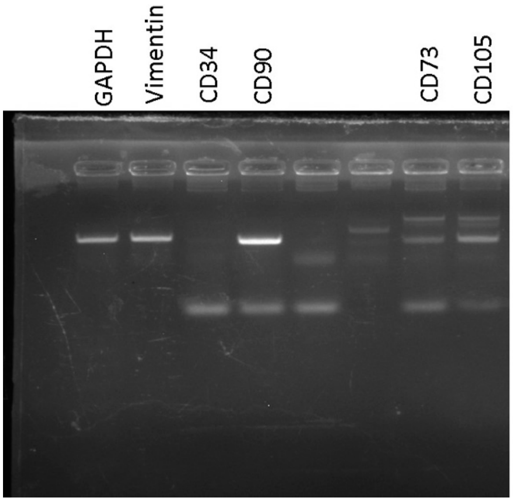

(a)

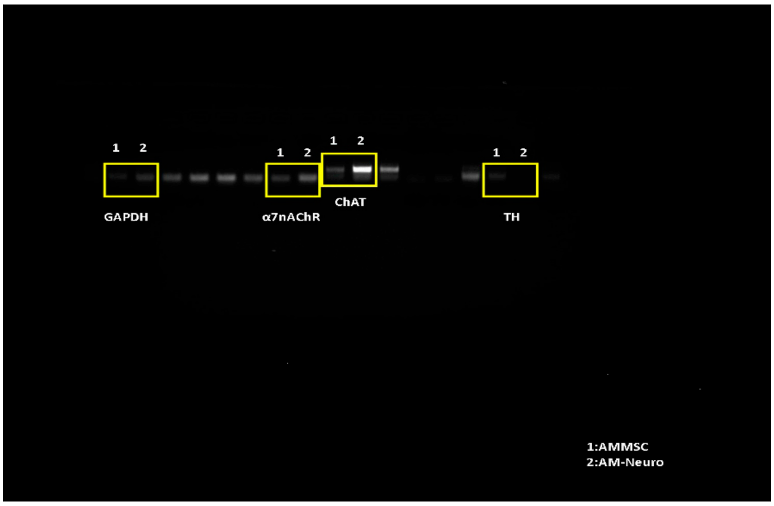

(b)

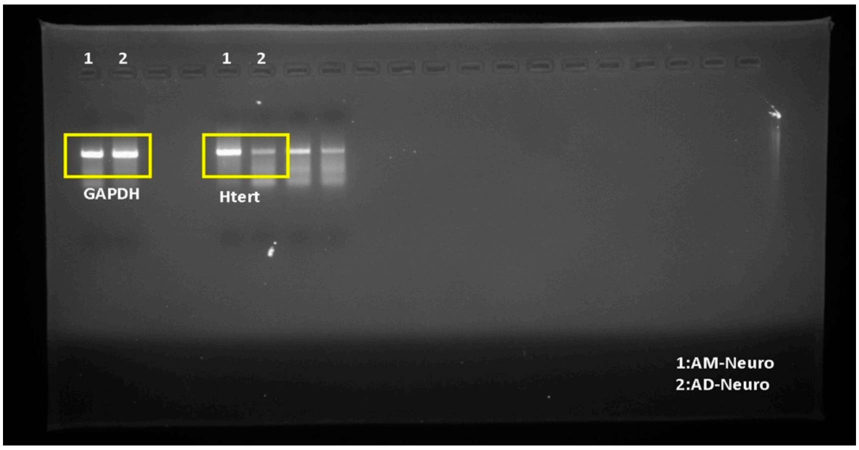

(c)

Data S2: Compilation of Western blots corresponding to Figure 3.

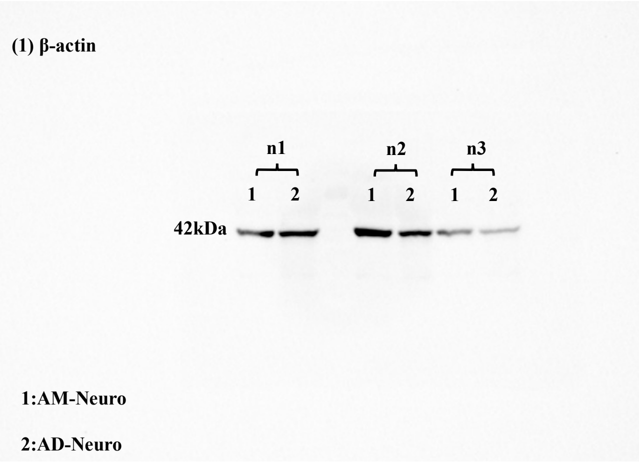
(1) β-actin
n1
n2
n3
1 2 1 2 1 2
42kDa
1:AM-Neuro
2:AD-Neuro

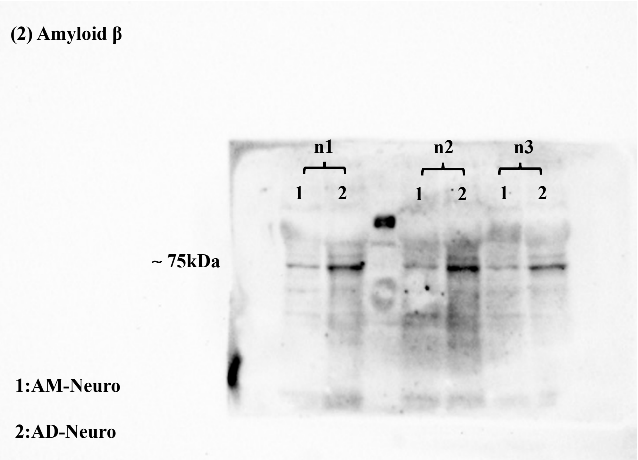
(2) Amyloid β
n1
n2
n3
1 2 1 2 1 2
~ 75kDa
1:AM-Neuro
2:AD-Neuro
